# ASO Author Reflections: Preoperative Selection of cT4b Esophageal Cancer Patients Who Benefit From a Salvage Robot-Assisted Minimally Invasive Esophagectomy (RAMIE)

**DOI:** 10.1245/s10434-020-09434-1

**Published:** 2020-12-02

**Authors:** Ingmar L. Defize, Sylvia van der Horst, Jelle P. Ruurda, Richard van Hillegersberg

**Affiliations:** 1 Department of Surgery, University Medical Center Utrecht, Utrecht, The Netherlands; 2 Department of Radiation Oncology, University Medical Center Utrecht, Utrecht, The Netherlands

## Past

Patients with esophageal cancer and clinical suspicion of invasion in adjacent irresectable structures such as the aorta and the tracheobronchial tree (cT4b) are usually precluded from surgery and treated with definitive chemoradiotherapy (dCRT) alone. dCRT often results in locoregional failure and a poor survival; therefore, treatment approaches to improve these outcomes in cT4b patients should be explored.

## Present

In the current report, a salvage robot-assisted minimally invasive esophagectomy (RAMIE) was assessed as a curative surgical approach for cT4b esophageal cancer following dCRT. In 92% (22/24) of patients, a radical resection was achieved, with a postoperative complication rate of Clavien–Dindo grade 2 or higher in 83% of patients. In these patients, the 12- and 24-month disease-free survival was 89% and 68%, respectively [1]. These favorable oncological results are attributable to a multimodality approach and the benefits of robotic surgery enabling meticulous dissection [2]. Other factors that are of significant importance to the success of this treatment strategy pertain to the proper selection of patients before surgery. In general, there are three aspects that should be taken into consideration during the preoperative course of cT4b patients.

First, a considerable proportion of patients (16%, 6/44) in whom a salvage RAMIE was deemed possible were omitted from surgery due to complications during or after dCRT that impeded an esophagectomy. Preventing complications such as esophageal perforation or the formation of fistulas to adjacent structures will allow more patients to proceed to and benefit from surgery.

Second, achieving a radical resection is pivotal to benefit from a salvage RAMIE. Together with previous reports, our results demonstrate that the survival of patients in whom an irradical resection is performed is dismal. A salvage RAMIE should therefore be reserved for patients in which sufficient downstaging is achieved and a radical resection is possible.

Third, in 46% of the included patients, a pathological complete response was achieved. These patients might have benefitted from an organ-sparing approach and could have been spared from the risk of perioperative complications; however, the risk on functional problems in an organ-sparing approach remains unclear and might outweigh its benefits.

## Future

The aforementioned challenges in the improvement of dCRT and proper patient selection can be tackled by a variety of strategies. Improvements in radiotherapy aid in the reduction of radiation damage to healthy tissue surrounding the esophagus, which may lead to a decrease in the formation of fistulas. One of these improvements is the recent implementation of the MRLinac, a linear accelerator combined with a magnetic resonance image (MRI) scanner, which allows radiation oncologists to irradiate with smaller margins and adapt radiation fields during dCRT, sparing healthy tissue from irradiation [3]. Additionally, the MRLinac enables the daily acquisition of MRI images during dCRT that provide a detailed overview of the thoracic anatomy. This enables the timely identification and active monitoring of fistulae formation or esophageal perforation.

To assess the resectability of the downstaged primary tumor during restaging, a detailed overview of the anatomical borders surrounding the esophagus should be acquired to assess persistent tumor invasion. In our center, a combination of modalities, including endobronchial ultrasound (EBUS), endoscopic ultrasound (EUS), positron emission tomography (PET) and (contrast-enhanced) computed tomography (CT) was used. All of these modalities provide valuable information but lack sufficient accuracy to independently assess resectability. Therefore, further research is warranted to increase the individual accuracy of these conventional modalities, to explore new modalities such as MRI, or to assess a multimodal diagnostic strategy that yields sufficient accuracy to determine resectability in downstaged cT4b esophageal tumors.

In order to safely offer an organ-sparing treatment to pathological complete responders, the results of currently running clinical trials on treatment response assessment of the primary tumor should be awaited [4]. A challenge, besides the assessment of local residual disease, will remain in the assessment of lymphatic spread after neoadjuvant treatment, which is of equal importance when considering an organ-sparing approach. Furthermore, when this approach can be offered safely, the functionality of the heavily irradiated esophagus and its effects on the quality of life will be of interest and should be assessed carefully.

To conclude, the aforementioned strategies will allow for an improved selection of patients with cT4b esophageal cancer amenable for curative treatment. These improvements could offer an increased number of patients with this condition a chance for cure and will ensure that salvage surgery is reserved for those patients who will benefit from this treatment.
